# Biomechanical Aspects of Actin Bundle Dynamics

**DOI:** 10.3389/fcell.2020.00422

**Published:** 2020-06-09

**Authors:** Julia Lange, Erik Bernitt, Hans-Günther Döbereiner

**Affiliations:** Institute of Biophysics, University of Bremen, Bremen, Germany

**Keywords:** actin bundles, filopodia, microcontact printing, filopodia angle, filopodia initiation, filopodia dynamics

## Abstract

Lamellipodial and filopodial protrusions are two of the main aggregate types of filamentous actin in living cells. Even though filopodia are essential to a range of vital cell functions, the mechanisms leading to their formation are still debated. Filopodia are relatively stiff and rod-like structures that are embedded in the highly dynamic framework of the backward flowing meshwork of the lamellipodium. Phenomena such as lateral filopodia drift and collision events suggest that mechanical aspects play a significant role in filopodia dynamics. In this paper, we systematically analyze the interplay between the backward flow of actin in the lamellipodium and the drift velocity of actin bundles, that we identify to be filopodia, in a quantitative manner in cells of given morphology and controlled myosin activity. Moreover, we study mechanical aspects of fusion of actin bundles drifting laterally in the lamellipodium. We find that the dynamics of actin bundles drift and fusion can be captured in a mechanical framework, which leads to a model of actin bundles orientation.

## Introduction

Filopodia are finger-like protrusions, consisting of crosslinked parallel bundles of actin filaments, which are often partly or completely embedded in branched actin networks of lamellipodia (Pollard and Borisy, 2003; Svitkina et al., 2003; Faix and Rottner, 2006). These highly dynamic cellular structures promote cell motility and play a fundamental role in cell navigation by sensing cellular environment and by adhering to extracellular matrixes (Steketee and Tosney, 2002; Gupton and Gertler, 2007; Mattila and Lappalainen, 2008). They are of special interest as they play a critical role in cancer cell invasion and metastasis (Machesky, 2008; Jacquement et al., 2015).

There has been considerable progress in identifying the protein composition of filopodia. Although the main players contributing to filopodia dynamics are identified, the processes involved in initiation of filopodia are being debated (Yang et al., 2007; Mattila and Lappalainen, 2008). Svitkina et al. (2003) proposed the “convergent elongation model,” where filopodial formation is initiated by reorganization of an Arp2/3 complex-nucleated dendritic network in the lamellipodium of mouse melanoma cells. In this framework, privileged bundles of actin filaments, which are protected by capping proteins, move laterally in the lamellipodium, collide with each other and fuse. In contrast, the “tip nucleation model” proposes nucleation of actin filaments at filopodia tips (Faix and Rottner, 2006; Ahmed et al., 2010). The latter model was inspired by studies showing formation of filopodia without a lamellipodium, via reducing the expression of the Arp2/3 complex (Steffen et al., 2006).

Lateral motion of filopodia has also been observed in matured filopodia of neuronal growth cones of *Aplysia* bag cell neurons. A study by Oldenbourg et al. (2000) concluded that lateral movement of radial actin bundles and filopodia occurs because of the interplay between actin polymerization at the tip of the bundle and retrograde flow of the actin network.

In addition to experimental studies, there are various theoretical mechanobiological approaches to explain the initiation and buckling process of filopodia. For example, simulations via a reaction-diffusion-elasticity system couple the membrane deformation to the organization of cortical actin to capture the filopodia initiation process (Isaac et al., 2013), whereas the limitations of filopodia length are calculated with Euler buckling of tightly linked filaments or with a membrane tube as an important stabilizer (Mogilner and Rubinstein, 2005; Pronk et al., 2008).

To bridge the understanding of molecular and mechanical processes, we have investigated the filopodia initiation process and their dynamics by analyzing experimental data and studying the behavior of moving actin bundles, that we identify to be filopodia, under changing biochemical conditions. One of the major drawbacks of studies is the limitation of measuring and comparing kinetic parameters such as actin bundles velocities or retrograde flow due to randomness of cell shapes and cell motility. To overcome these issues, we create circular fibronectin patches, which force fibroblasts to fixed morphologies using artificial constraints to measure the response of the system.

###  Embedding Model of Actin Bundles Motility and Fusion in Disk-Shaped Fibroblasts

We present characteristic distributions of kinetic parameters of movement of actin bundles as a function of myosin II motor activity using a known inhibitor of myosin II to affect retrograde actin flow (Kovács et al., 2004; Parsons et al., 2010). In detail, we measured systematically lateral velocities of actin bundles, retrograde actin flow and angles of actin bundles, enabled by the microcontact printing and consistent morphologies of the cells. We find slower lateral velocities of actin bundles to be induced by decreased retrograde actin flow. Whereas, the main sources driving retrograde actin flow are identified (Henson et al., 1999), the origin of angles of actin bundles is largely unknown. We propose that there are three different interactions or properties restricting actin bundles to grow under defined angles: (i) required polymerization energy of actin bundles varies with the angle, (ii) embedding of actin bundles in the lamellipodium limits angles of actin bundle's tips, and (iii) interaction of actin bundles with undulations in the lamellipodium (actin folds) changes actin bundle's angles.

Moreover, we analyze the fusion process of actin bundles in disk-shaped cells. We explain how tips of two actin bundles come into contact and merge. Locally, there are twice the number of free filament ends at a constant concentration of actin monomers. This leads to a thinning of the newly formed actin bundle, resulting in its buckling and re-embedding.

## Materials and Methods

###  Cell Culture

Mouse embryonic RPTP α^+/+^ fibroblasts were cultured in Dulbecco's modified Eagles medium (DMEM, Biochrom) containing 100 μg ml-1 Penicillin/Streptomycin (PAA) and 10 % Fetal Bovine Serum (Biochrom) at 37 °C and 5 % CO_2_.

To knockdown myosin X, fibroblasts were transfected with a siRNA with the myosin X specific sequence CGACGGCGACUAUGACUAUtt using Lipofectamine2000 according to the manufacturer's protocol (Invitrogen). We detected the time at which the effect of myosin X knock-down was maximal in cells on overall fibronectin-coated glass coverslips. Thus, 25 fibroblasts were monitored over 48 h after transfecting the cells (data not shown). A decrease in the number of actin bundles was observed over time until a minimum with a nearly complete inhibition of actin bundles initiation after 24 h was reached. Afterwards an increase in the number of actin bundles was detected and after 48 hours nearly the same number of actin bundles per cell as in control cells were detected. Based on this result, we plated transfected cells on microcontact printed substrates 24 h after transfection.

To inhibit myosin II, cells were treated with Blebbistatin (Sigma-Aldrich). Thus, cells adhered on microcontact printed substrates for 20 min. Afterwards, DMEM was replaced by DMEM with Blebbistatin (25 μM), and these cells are denoted as Blebbistatin treated cells in this study. The concentration of 25 μM was found as an appropriate concentration at which an effect of an interference with the myosin II-machinery was observed. In phase contrast images it appeared in form of a larger growing lamellipodium (see Figure S2) in contrast to untreated cells. The concentration of 25 μM was set as the optimal working point as cells treated with this concentration showed a high response, whereas cells treated with higher doses of Blebbistatin exhibited substantial disruption of the cytoskeleton.

Cells were stained with Vybrant DiI (Molecular Probes) according to the manufacturer's protocol for visualization of the cell membrane with total internal reflection fluorescence (TIRF) microscopy.

###  Microcontact Printing

To shape cells into disk-like morphologies, we used microcontact printing. Following a protocol by Théry and Piel (2009), we fabricated stamps out of polydimethylsiloxan (PDMS, Sylgard 184 silicone elastomere) as a cast. It contained disk-like structures with areas of 4.000 μm^2. Glass coverslips were activated with a plasma source Kinpen 11 (Neoplas Control). Disk-like fibronectin (Roche, 50 μg ml-1 diluted in PBS) patches were printed (with the PDMS stamps) on the activated glass coverslips. The areas between fibronectin patches were coated with PLL-g-PEG (SuSoS Surface Technology, 100 μg ml-1 diluted in 10 mM HEPES buffer) to prevent cell adhesion. In prior experiments, it has been shown, that fibronectin adhesion patterns of 4.000 μm^2 are of an appropriate size, so that only one cell can adhere at one patch (data not shown). Larger patch areas led to an attachment of two or more cells per patch.

###  Sample Preparation and Live Cell Imaging

Cells were seeded in petri dishes with microcontact printed substrates. After 20 min of incubation, cells were rinsed with phosphate buffered saline (PBS) to wash out non-adhered cells and fresh DMEM was added.

We used a Zeiss Axio Observer.Z1 equipped with a Heating Unit XL S, a Temp Module S, a Pecon Heating Insert P S1 and a CO_2_ Module S to generate physiological conditions of 37 °C and 5 % CO_2_ for live imaging. Phase contrast microscopy, using a 10x objective with a numerical aperture of 0.25, enabled a stable focus for long-term experiments and was used for all experiments of this study, but the ones carried out using TIRF illumination.

For TIRF microscopy, cells were stained with Vybrant DiI (Molecular Probes). A laser of 561 nm wavelength (part of a Zeis TIRF system) and a 100x objective with a numerical aperture of 1.46 were used. TIRF was used for monitoring the spreading phase of the cells and especially for a higher resolution of dynamics of actin bundles in the late spreading phase.

###  Measuring Distributions of Lateral Actin Bundles Velocity, Angle, and Retrograde Actin Flow

For visualization of lateral movement of actin bundles and lateral velocity measurements (see **Figure 2B**), we created one-dimensional circular kymographs with FIJI (Schindelin et al., 2012; Bernitt et al., 2015). In these space-time-plots, intensities from phase contrast images of equal angular segments along an arc with a width of one pixel, averaged radially at the cell edge, were plotted against time (**Figure 4D**).

To measure retrograde actin flow velocity perpendicular to the membrane edge into the cell, we calculated one-dimensional autocorrelation functions of kymographs. These kymographs are space-time plots of intensities from phase contrast images of the lamellipodium perpendicular to the cell edge over time using Matlab2013b (The Mathworks) (Döbereiner et al., 2006). This means autocorrelation function of the retrograde actin flow from different time points at the same location along the cell edge were calculated. These measurements were repeatedly performed at different locations in the surrounding area of moving actin bundles. Mean values were calculated that represents one data point (**Figure 2A**).

We obtained these autocorrelation functions at different contour points in between interaction loci of different actin bundles and calculated a mean value in order to get characteristic values of retrograde actin flow velocity. Using this technique allows to obtain detailed distributions revealing quantitative functional dependencies as a function of biochemical conditions.

For measuring angles between actin bundles main axis and the normal of the cell membrane, we used the angle tool in FIJI. We calculated skewness of angle distributions as the third standardized moment.

## Results

###  Disk-Shaped Cells Allow a Systematic Quantitative Analysis of Movement of Actin Bundles

**Figure 1:**
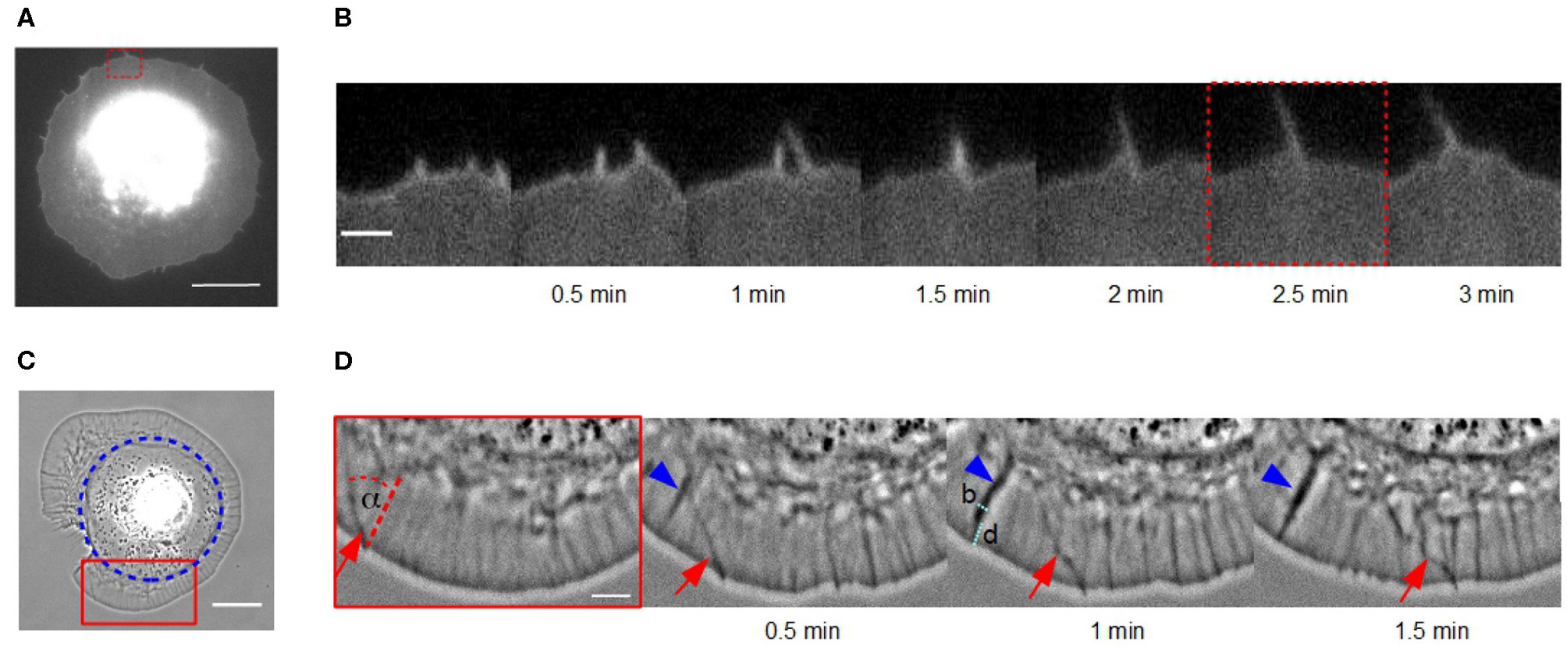
Lateral movement of actin bundles in fibroblasts on homogeneous substrates vs. disk-like patches. **(A)** Micrograph of a cell membrane-stained (DiI) fibroblast in the late spreading phase on a homogeneous fibronectin coated glass coverslip using TIRF microscopy (scale bar: 15 μm). **(B)** Time-lapse sequence of the red dashed region of interest in **(A)** (scale bar: 2 μm). **(C)** Phase contrast micrograph of a disk-shaped fibroblast (scale bar: 20 μm). Blue circle indicates the fibronectin patch on which the cell adhere. **(D)** Time-lapse sequence of the red solid region of interest in **(C)**. An actin bundle, which moves under angle α perpendicular to the cell membrane is highlighted by red arrows (scale bar: 5 μm). Blue arrowhead indicates an actin fold with their width *b* and distance from the cell membrane *d*.

Dynamics of actin bundles were observed in fibroblasts on homogeneous fibronectin-coated glass coverslips. Figures 1A,B show a membrane-stained fibroblast during its late isotropic spreading phase on a homogeneous substrate using TIRF microscopy. During this phase, actin bundles move laterally along the cell membrane, colliding, fusing and forming protrusions of a length of several micrometers. This fusion and growth process is shown in detail in Figure 1B and was initially captured in the convergent elongation model (Svitkina et al., 2003).

We also observed actin bundles in disk-shaped fibroblasts on fibronectin patches (Figure 1C). This system enables an analysis of movement of actin bundles in nearly perfect disk-shaped cells at least for 30 min. In contrast, the spreading phase of fibroblasts on homogeneous substrates is not suitable for analyzing filopodia dynamics because the cell shape is altering over the whole time.

The small actin bundles seem to be post-precursors of filopodia with a maximal length of one micrometer and they only move laterally in two dimensions. In this system, we do not observe larger actin bundles. To verify that these laterally moving bundles in fibroblasts on fibronectin patches are filopodia and to distinguish them from passive mere actin bundles, we performed a control experiment and investigated the influence of knockdown myosin X on them. It is known that filopodia formation requires myosin X (Tokuo and Ikebe, 2004; Tokuo et al., 2007; Bear and Gertler, 2009). Twenty-four hours after transfecting cells with siRNA, we did not find laterally moving bundles of actin filaments in myosin X depleted fibroblasts (see Figure S1). Thus, these structures could be identified as filopodia.

It seems, that the appearance of actin bundles was not affected by the restricted morphology of cells on microcontact printed substrates, and collision and fusion still occurred (Figure 1D). However, the exploration radius (*x* ≈ 0.3 μm), defined as the distance between the cell membrane and the tip of the actin bundle, and the length of a actin bundle (*l* ≈ 1 μm) were several micrometers shorter. Instead of growing long distances, actin bundles in cells of restricted morphology buckled and broke before continuing to move at a changed angle.

Moreover, we observed a number of actin folds in disk-shaped fibroblasts that have not been mentioned before in the literature (Figure 1D). These are undulations in the lamellipodium, which are not formed directly at the cell edge but rather at a distance *d* from the leading cell membrane (Figure 1D). They are conjectured to be caused by a curved membrane comparable to wrinkling patterns in soft shells and soft bilayers (Shao et al., 2016; Zhang et al., 2018). Since, we have incompressible material, the disk-shaped system reacts with wrinkling to inward bound radial flow. They are not directly the focus of this study but influence actin bundles dynamics. When actin bundles move through these folds, they seem to interact elastically with them and the orientation of actin bundles changes.

###  Inhibiting Myosin II Activity Leads to a Slowing Down of Lateral Velocities of Actin Bundles Coupled to a Downward Shift and Narrowing of Retrograde Flow Distribution

We focused on stationary cells on fibronectin patches that exhibited a round and smooth lamellipodium directly after spreading. Data were systematically gathered in form of phase contrast images in order to measure distribution functions of lateral velocities of actin bundles, retrograde actin flow and tilt angle of actin bundles. We reduced retrograde actin flow using Blebbistatin with a concentration of 25 μM. The latter inhibits myosin II, which drives retrograde actin flow (Lin et al., 1996; Kovács et al., 2004; Anderson et al., 2008).

**Figure 2:**
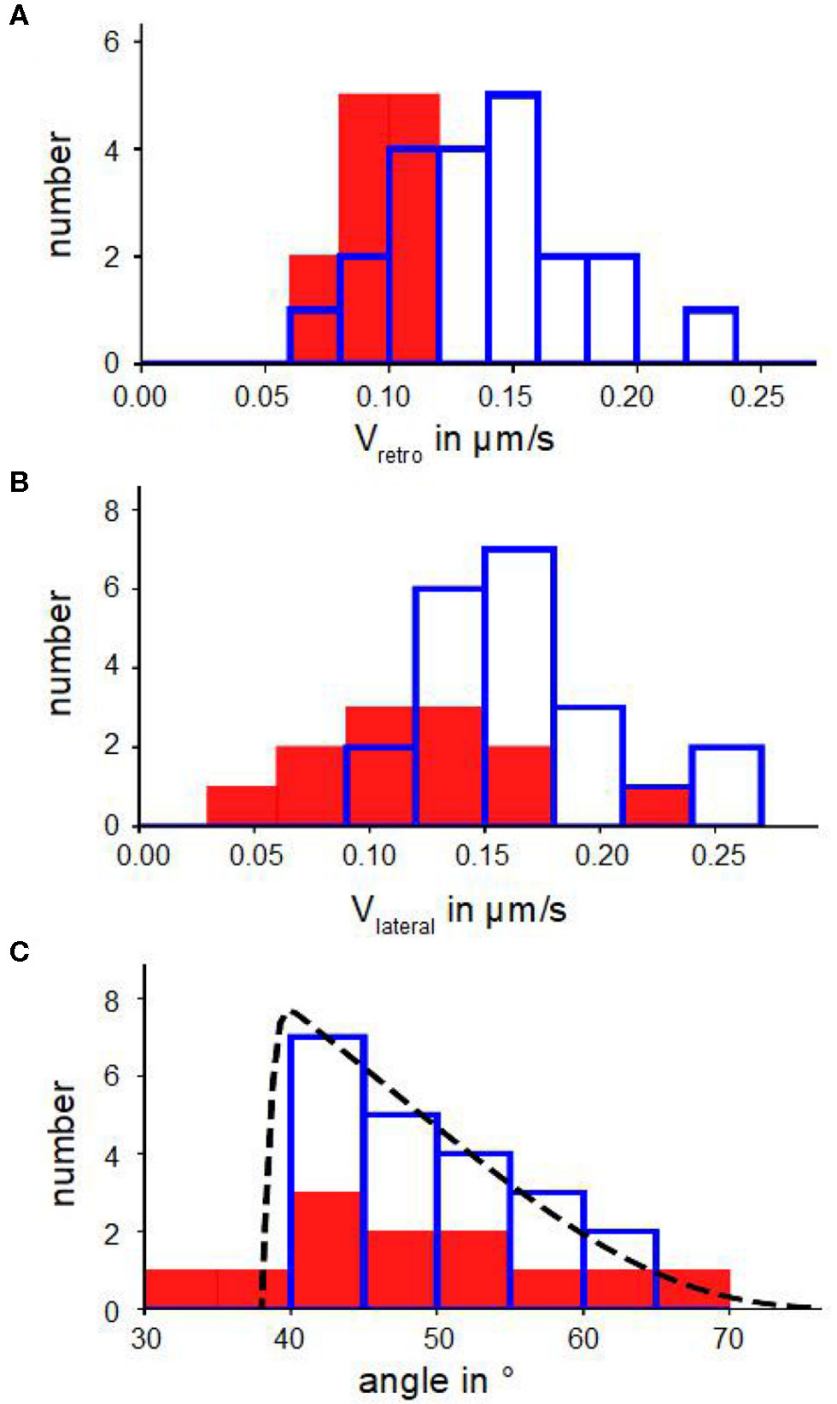
Kinetic parameters of actin bundles. **(A)** Histograms of retrograde actin flow. **(B)** Histograms of lateral velocities of actin bundles. **(C)** Angle distributions with a fit n(α) (Equation 6). In **(A–C)** blue bars show number of actin bundles in 10 disk-shaped cells without Blebbistatin, red filled bars represent number of actin bundles in 7 disk-shaped cells treated with Blebbistatin (25 μM).

We discovered (i) a reduced retrograde actin flow velocity (Table 1) and (ii) a much smaller distribution width of retrograde actin flow values in Blebbistatin treated cells when compared to untreated cells (Figure 2A). The shift to lower flow velocities is due to the fact that retrograde actin flow is driven by two different mechanisms: on the one hand, actin polymerization against the membrane creates a rearward actin flow. On the other hand, myosin II is involved in contraction and pulling back of actin filaments which leads to an additional flow component (Henson et al., 1999; Parsons et al., 2010; Craig et al., 2012). By using Blebbistatin, we inhibited the myosin II-driven part of retrograde actin flow. The contribution of retrograde actin flow following from actin polymerization against the cell membrane should be unaffected.

**Table 1:** Mean values μ and standard deviation σ of lateral velocities and retrograde flow velocities in untreated disk-shaped cells (*n* = 21) and disk-shaped cells treated with 25 μM Blebbistatin (*n* = 12).

	**Untreated cells**		**Blebbistatin**	
	**μ/μm s^-1^**	**σ/μm s^-1^**	**μ/μm s^-1^**	**σ/μm s^-1^**
Retrograde flow	0.14 ± 0.01	0.04	0.09 ± 0.01	0.02
Lateral velocity	0.16 ± 0.01	0.04	0.12 ± 0.01	0.05

**Figure 3:**
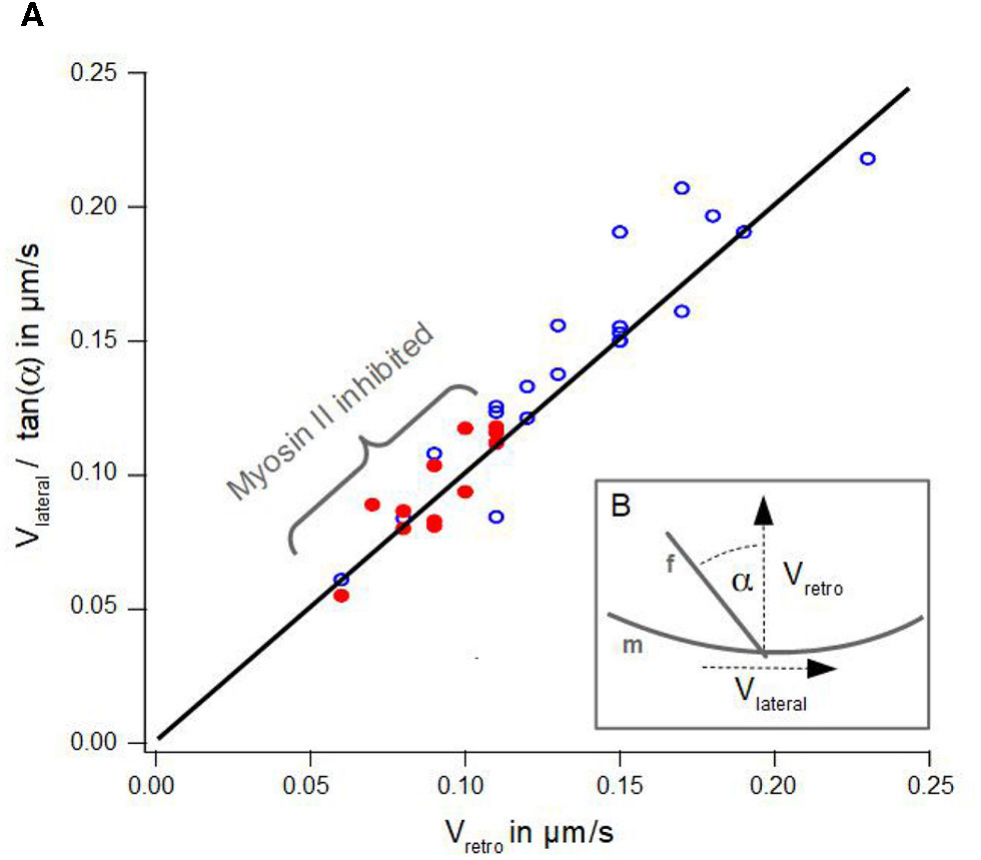
Geometrical Coupling. **(A)** Lateral velocity divided by angle of an actin bundle is plotted against retrograde actin flow. Blue dots show data of disk-shaped cells without Blebbistatin, red dots visualize data of disk-shaped cells treated with Blebbistatin (25 μm). Solid line visualizes a line from the origin with a slope of 1 after Equation 1. **(B)** Schematic illustration of measured lateral actin bundles velocity *v*_lateral_, retrograde actin flow *v*_retro_ and tilt angle α of an actin bundle f with respect to the membrane m.

Figures 2B,C show distributions of lateral velocities of actin bundles and angles of actin bundles respectively. We noticed a clear triangular shape of the angle distribution. We found a skewness of γ=0.40 in untreated cells and γ=0.53 in cells treated with Blebbistatin (Table 2), that captured the asymmetry. Note that in contrast to a former study (Oldenbourg et al., 2000), we measured lateral velocities parallel to local membrane orientation and angles of actin bundles with respect to the normal of the cell membrane (Figure 3B).

**Table 2:** Mean values μ, standard deviation σ, and skewness γ of actin bundles angles in untreated disk-shaped cells (*n* = 21) and disk-shaped cells treated with 25 μM Blebbistatin (*n* = 12).

	**Untreated cells**			**Blebbistatin**		
	**μ/^°^**	**σ/^°^**	**γ**	**μ/^°^**	**σ/^°^**	**γ**
Angle	49 ± 1	6	0.40	49 ± 1	10	0.54

We verified the statistical significance of the difference between the mean lateral velocities of normal and Blebbistatin-treated cells using a two-sided Mann–Whitney *U*-test. We found that the reduction of the mean lateral velocities of cells treated with Blebbistatin is statistically significant at 5%. However, mean value and distribution of tilt angle of actin bundles are similar in Blebbistatin treated cells and in untreated cells (Figure 2C).

Figure 3A summarizes the data of lateral velocities of actin bundles, retrograde actin flow velocities and tilt angles of actin bundles in cells treated with Blebbistatin and untreated cells. The lateral velocity of actin bundles *v*_lateral_ divided by tan(α) is plotted as a function of the retrograde actin flow velocity *v*_retro_.

According to Oldenbourg et al. (2000), viewing the actin bundles as embedded into the lamellipodium (Svitkina et al., 2003), the lateral velocity *v*_lateral_ depends on the retrograde actin flow velocity *v*_retro_ and the tilt angle α of the actin bundle (Figure 3):

(1) $$\begin{array}{r} |v_{\text{lateral}}|=|v_{\text{retro}}|\cdot \text{tan}\left(\alpha \right). \end{array}$$

However, we noted a slight deviation in our data. Indeed, we found a small lateral velocity *v*_0_ greater than zero at the point where no retrograde actin flow exists. We conjecture even in the absence of retrograde flow, that lateral movement of actin bundles is induced by bending of elongating filaments already in contact with the membrane at equilibrium tension.

Additionally, graph 3A visualizes a remarkable shift along the linear regression line between data points from cells treated with Blebbistatin and data points from untreated cells. This is a consequence of a reduction of retrograde actin flow velocities and their much smaller distribution width (Figure 2A).

The analysis of Oldenbourg et al. (2000) was performed in growth cones of *Aplysia* bag cell neurons. We noted that our data differ significantly in the range of angles of actin bundles. Neuronal growth cones are quite long and almost perpendicular to the membrane. In contrast, we found a much wider distribution and the absence of small angles in actin bundles.

###  Actin Bundles Fusion Process in Disk-Shaped Cells

**Figure 4:**
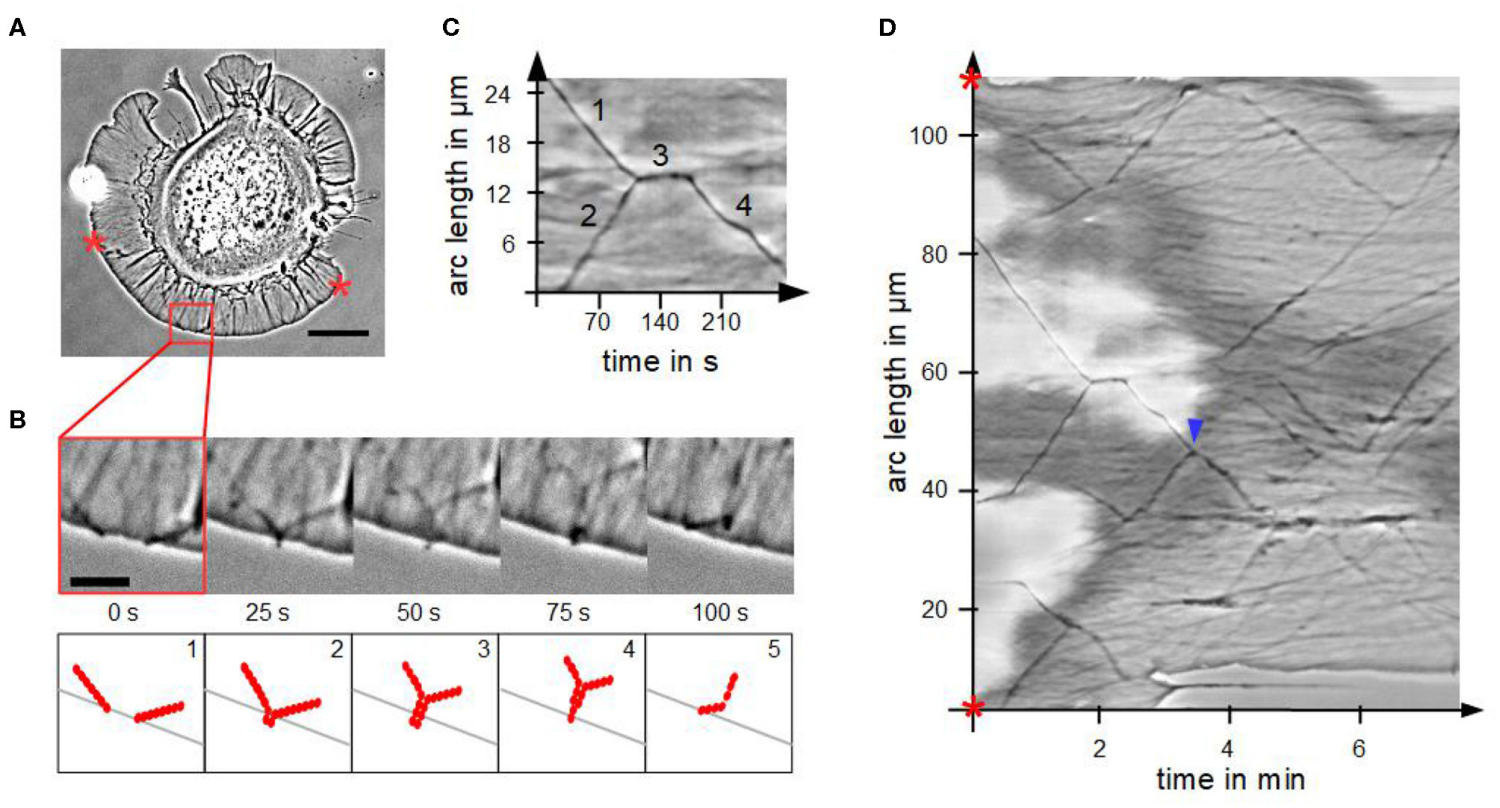
Fusion of actin bundles. **(A)** Phase contrast micrograph of a disk-shaped fibroblast attached to a substrate with lateral moving bundles of actin filaments (scale bar: 20 μm). **(B)** Time-lapse sequence of the red region of interest in **(A)** (scale bar: 5 μm) and schematic illustration of the fusion process of actin bundles. The process occurs in five steps: 1. lateral movement of several actin bundles, 2. actin bundle's tips come into contact, 3. elongation, 4. thinning and 5. buckling of the actin bundle. **(C)** Cutout of the kymograph D visualizing a stationary fusion of two actin bundles. **(D)** Circular kymograph of the section between two stars in **(A)** offers an overview of movement and fusion of various actin bundles. Blue arrowhead highlights a “mobile fusion”.

We analyzed the fusion process of actin bundles in disk-shaped fibroblasts, which is visualized in Figures 4A,B. Fusion started when tips came into contact in a zipper-like manner, which usually lasted for several seconds. By means of a kymograph (Figures 4C,D), we determined interesting characteristics and parameters of fusion of actin bundles. The cutout in Figure 4C from the kymograph of Figure 4D visualizes the mechanism of a stationary fusion: at the beginning two actin bundles moved laterally (1 and 2). They crossed, fused and remained stationary for 30 s (3). Afterwards the newly created actin bundle moved laterally (4) with a different velocity than the individual actin bundles before their fusion, which coincided with a changed angle. The actin bundles exhibit conserved length, which is in contrast to the continual rise of length of actin bundles in randomly spread cells.

Not in all cases the fusion of two actin bundles included a temporal stationary phase. In these so-called “mobile fusions” (Figure 4D), there was no stationary part after tips of actin bundles came into contact. The tips fused during locomotion of the two actin bundles in one direction.

## Discussion

In the following we emphasize the mechanobiological features of embedded actin bundles. First, we model fusion of actin bundles by considering molecular and mechanical processes. Second, we explain the skewed actin bundles angle distributions by pure mechanics considering interactions of embedding actin bundles with actin folds and the cell membrane as well as the influence of the fusion process.

###  Kinetics of Actin Bundles and Their Fusion Are Affected by an Imbalance of Availability of Actin Monomers and Membrane Tension

The model of fusion of actin bundles (Figure 4B) describes how two actin bundles tips come into contact. This leads to the occurrence of twice the number of free filament ends in a tip at a constant g-actin concentration, resulting in buckling and re-embedding of a newly formed actin bundle.

In detail, at the beginning, actin bundles move toward each other at defined angles (Figure 4B1). The movement occurs due to the interplay between actin polymerization at the tips of actin bundles and retrograde actin flow of the actin meshwork (Oldenbourg et al., 2000). The length of each actin bundle is limited by buckling because of membrane resistance (Mogilner and Rubinstein, 2005). This means a defined number of actin filaments has to be bundled to protrude the membrane. When the tips of two bundles of actin filaments come into contact, the bundles merge like a zipper (Svitkina et al., 2003) (Figure 4B2). More specifically: the actin bundle's tips interlink before the actin bundles merge over the whole length. At the beginning of this process, locally there are double as many barbed ends of actin filaments at actin bundle's tips where actin monomers can polymerize. Due to the larger number of actin filaments, which mechanically stabilizes the actin bundle, the fused actin bundle can grow slightly longer as it can compensate more deformation energy of the membrane (Figure 4B3).

The increased number of filaments locally disturbs the equilibrium between g-actin monomer availability and number of growing filament ends. From a theoretical model, in which G-actin diffusion limits the length of thick actin bundles (Mogilner and Rubinstein, 2005), it can be concluded, that locally, there are not enough monomers present to polymerize at twice the number of filament ends (Figure 4B4). The actin bundle will become thinner after a period of growth because polymerization only occurs at some, rather than at all filaments. As a result, the actin bundle buckles due to its length and thin shape. Consequently, this causes a renewed movement of the created actin bundle under a defined angle (Figure 4B5).

This leads to the question about the cause of different actin bundles lengths in disk-shaped fibroblasts in contrast to randomly spread fibroblasts. A possible explanation is a difference in membrane tension, positioning of adhesions and retrograde actin flow. We measured a mean retrograde actin flow of 0.14 μm s-1 (Figure 2B) in fibroblasts on microcontact printed substrates. In comparison, retrograde actin flow in Swiss 3T3 mouse fibroblasts, REF-52 rat fibroblasts and B16 mouse melanoma cells ranged from 0.01 μm s-1 to 0.12 μm s-1 (Alexandrova et al., 2008). Contrary to randomly spread cells, fibroblasts on fibronectin patches built large lamellipodia growing beyond the substrates. The areas between the substrates were filled with PLL-g-PEG, on which the cells cannot adhere. The limited space on disk-like fibronectin patches and arrangement of adhesions is coupled to a lower membrane tension (Pontes et al., 2017) and faster retrograde actin flow (Parsons et al., 2010; Yamashiro and Watanabe, 2014) in cells on disk-like fibronectin patches which can be associated with buckling of actin filaments.

###  Mechanobiological Interactions Leads to a Skewed Distribution of Actin Bundles Angles

In the following, we derive a physical model, which is in very good agreement with the triangular shaped angle distribution in Figure 2C. We hypothesize that there are three effects causing agles of actin bundles:

1. actin bundles require more polymerization energy to grow under larger angles,
2. actin bundles are embedded in the lamellipodium (Svitkina et al., 2003) and
3. actin bundles interact with actin folds in the lamellipodium.

In detail, actin polymerization at actin bundle's tips generates a force *f* along their length against the membrane:

(2) $$\begin{array}{r} f=\tau \cdot l. \end{array}$$

The length of an actin bundle is divided into the part, which is embedded in the lamellipodium and the part *l* which marks the visible outward finger-like protrusion. The membrane tension τ is independent of actin bundle's angle, whereas the length of an actin bundle increases with the angle. Polymerization energy to build up an actin bundle and the required work to overcome the membrane tension (*E*) depends on a pushing force perpendicular to the membrane *f*_⊥_:

(3) $$\begin{array}{r} E=\frac{x\cdot f_{⊥}}{\text{cos}\left(\alpha \right)}. \end{array}$$

As a consequence, it requires more polymerization energy *E* to build up actin bundles under larger angles at a constant exploration radius *x* of the cell. Assuming a Boltzmann distribution, this leads to a suppression of actin bundles, that grow under large angles, proportional to $e^{-\frac{a}{\text{cos}\left(\alpha \right)}}$. Exploration radius *x*, pushing force against the membrane *f*_⊥_ and thermal energy *k*_*B*_ · *T* are conflated in the constant *a*:

(4) $$\begin{array}{r} a=\frac{x\cdot f_{⊥}}{k_{B}\cdot T}. \end{array}$$

The second assumption is that actin bundles are embedded in the dendritic network of a lamellipodium and are sharing its symmetry (Svitkina et al., 2003). Pollard et al. found Y-shaped branches due to accumulation of Arp2/3-complexes with characteristic angles of 70° (Pollard and Borisy, 2003), which means that actin filaments cannot grow under angles much smaller than 20° with regard to the membrane. This is approximately in line with electron micrograph analysis of angle distributions of individual filaments (Koestler et al., 2008) and leads to the conclusion that in our system actin bundles angles greater than 70° with regard to the normal of the cell membrane are suppressed.

Moreover, we observed actin folds, that are proposed to be caused via topological constraints of a centripetally contracting, curved membrane (Figure 1D). Embedded actin bundles interact elastically with the ensuing folds. Actin bundles with small angles are suppressed because they have to be strongly curved by passing actin folds that require bending energy. Actin bundles with larger angles can grow without hindrance. Angles of actin bundles α are determined by the ratio of the actin folds width *b* and the distance from the membrane *d*:

(5) $$\begin{array}{r} tan\left(\alpha \right)=\frac{b}{d}. \end{array}$$

It should be noted, that the shape of the angle distribution is independent from the fusion process. After fusion of two actin bundles, the newly formed actin bundle moves under a different angle, but this actin bundle will also be embedded according to the underlying symmetry as described above.

Based on this, we can write down the angle distribution *n*(α) of untreated disk-shaped cells:

(6) $$\begin{array}{r} n\left(\alpha \right)=c\cdot e^{-\frac{a}{\text{cos}\left(\alpha \right)}}\cdot \text{erf}\left(\frac{\alpha -\alpha_{0}}{\Delta \alpha }\right). \end{array}$$

Setting $\alpha_{0}=38^{∙}$ and Δα = 1° by visual inspection of the sharp cut-off at small angles, a least square fit of the distribution (Equation 6) returns a parameter *a* = 2 with a normalization factor *c* (Figure 2C). We calculate a pushing force of the actin bundle perpendicular to the membrane of *f*_⊥_ = 0.03 pN using Equation (4) with an exploration radius of *x* = 0.3 μm.

The calculated pushing force in these experiments is three order of magnitudes smaller than in theoretical approaches (*f* ≈ 10 pN, as described in Mogilner and Rubinstein, 2005; Dmitrieff and Nédélec, 2016). In contrast, experimental studies measured smaller pushing forces of single actin filaments and small bundles of actin filaments of *f* ≈ 1 pN (Footer et al., 2007). The smaller pushing force in our experiments might result from a lower membrane tension. This follows from different geometries and positioning of adhesions in these experiments compared to random spread cells.

## Conclusion

This article exposes that purely mechanical reasoning explains the dynamics of lateral actin bundles drift in the lamellipodium and that of actin bundles collision events. We showed via experimental control of the lamellipodial retrograde flow that the moving frame of reference of the lamellipodium, in which the actin bundles are embedded, is the major effector of lateral actin bundles motion. The geometric constraints of filament orientation and length, as well as the mechanical tension of the plasma membrane act as additional control variables. Our results demonstrate the important role of mechanobiology with the physics of actin bundles angle distribution being a paradigmatic example. We consider the absence of dynamical actin bundles, which have the morphological appearance and spatio-temporal characteristics of filopodia, trggered by the selective biochemical response to myosin X knockdown, as a strong indication for the identity of these structures as filopodia.

## Data Availability Statement

All datasets generated for this study are included in the article/Supplementary Material.

## Author Contributions

The experiments were designed and analyzed by JL, EB, and H-GD. They were performed by JL and EB. The paper was written by JL, EB, and H-GD.

## Conflict of Interest

The authors declare that the research was conducted in the absence of any commercial or financial relationships that could be construed as a potential conflict of interest.
