# Supplementary material for: Biomechanical Aspects of Actin Bundle Dynamics
Source: Front Cell Dev Biol. 2020 Jun 9;8:422. doi: 10.3389/fcell.2020.00422 (PMC7296148; doi:10.3389/fcell.2020.00422)
Supplement: Supplementary file 1 [file Image_1.pdf]

Supplementary Material

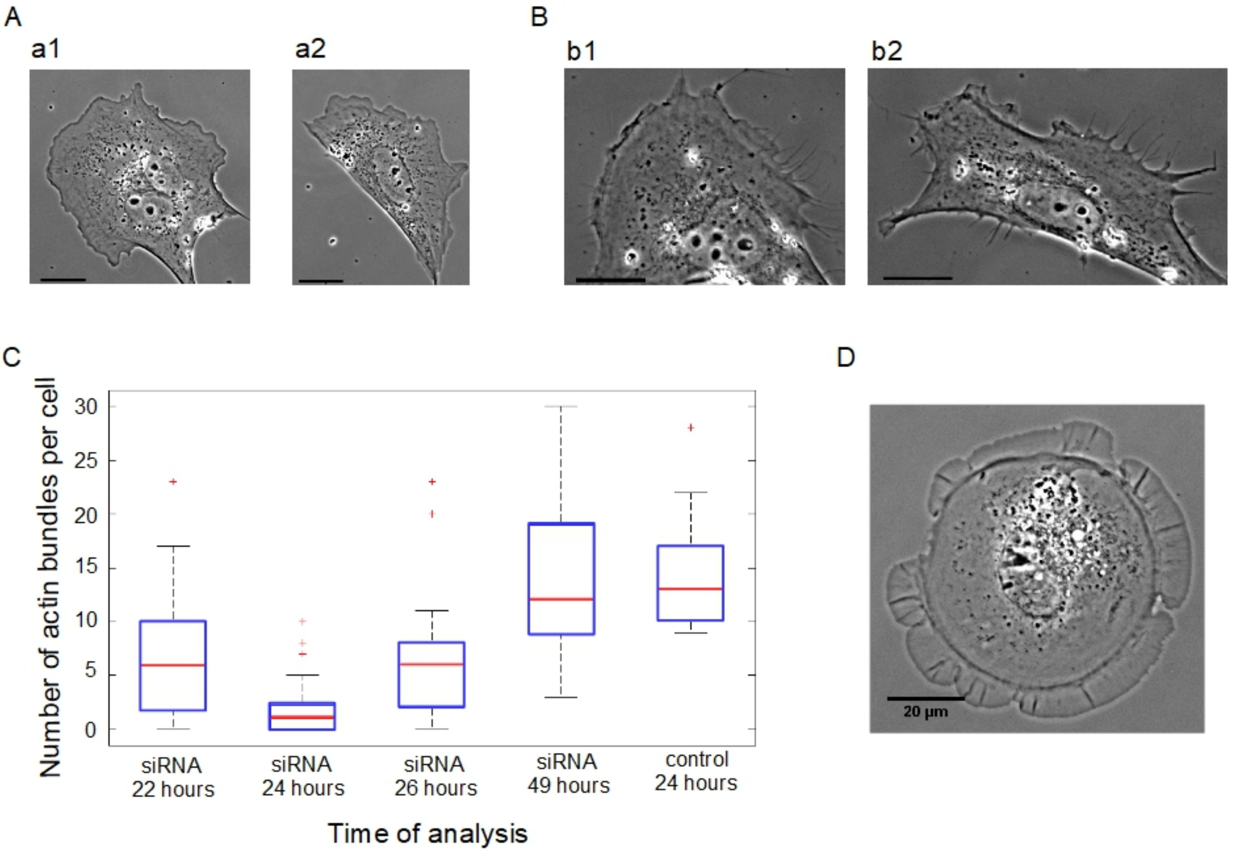

Figure S1. A: examples of random-shaped fibroblasts 24 hours after transfection with siRNA to knockdown myosin X. B: examples of randomly shaped control cells at the same time point. Scale bars: 20 µm. C: boxplot visualizes the number of actin bundles (here: actin bundles are a generic term including retraction fibres and filopodia) in myosin X knockdown cells 22, 24, 26 and 49 hours after transfecting them with siRNA as well as control cells after 24 hours. D: example of a disk-shaped fibroblast 24 hours after transfection with siRNA. There are no actin bundles in the lamellipodium after a knockdown of myosin X.

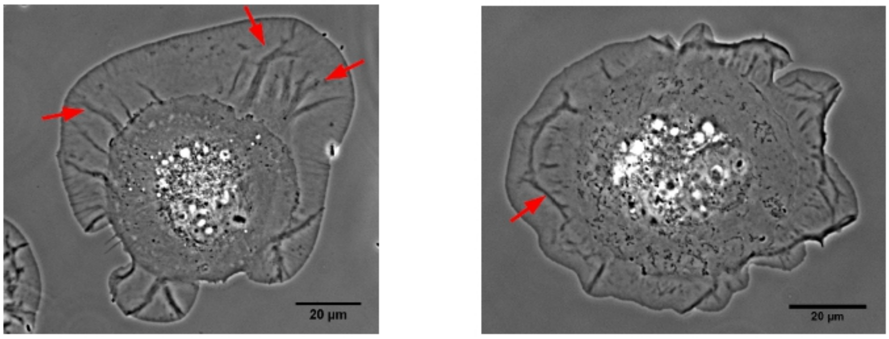

Figure S2. Two examples of fibroblasts on adhesion patterns 30 minutes after adding 25 µM Blebbistatin to inhibit myosin II. One can observe excessively large grown lamellipodia. Red arrows indicate actin folds in cells treated with Blebbistatin.
